# BCAR1 promotes proliferation and cell growth in lung adenocarcinoma via upregulation of POLR2A

**DOI:** 10.1111/1759-7714.13676

**Published:** 2020-10-01

**Authors:** Chun‐guo Mao, Sha‐sha Jiang, Cheng Shen, Tan Long, Hua Jin, Qun‐You Tan, Bo Deng

**Affiliations:** ^1^ Thoracic Surgery Department, Institute of Surgery Research Daping Hospital, Army Medical University Chongqing China

**Keywords:** BCAR1, cell growth, cell proliferation, lung adenocarcinoma, POLR2A

## Abstract

**Background:**

This study was designed to investigate the effects of a novel carcinogenetic molecule, p130cas (breast cancer antiestrogen resistance protein 1 or BCAR1) on proliferation and cell growth in lung adenocarcinoma. The study also aimed to identify the possible underlying signal networks of BCAR1.

**Methods:**

First, we evaluated proliferation, cell colony formation, apoptosis, and cell cycle after BCAR1 was knocked out (KO) using CRISPR‐Cas9 technology in H1975 and H1299 human lung adenocarcinoma cells. Subsequently, BCAR1 was upregulated in 293T cells and immunoprecipitation‐mass spectrometry (IP‐MS) was used with bioinformatics analysis to screen for potential networks of BCAR1 interacting proteins. Ultimately, we validated the correlated expressions of BCAR1 and a selected hub gene, RNA polymerase II subunit A (POLR2A), in 54 lung adenocarcinoma tissues, as well as in H1975 and H1299 cells.

**Results:**

Cell proliferation of H1975 and H1299 was significantly inhibited following BCAR1‐KO. Colony formation of H1975 cells was also significantly decreased following BCAR1‐KO. IP‐MS demonstrated 419 potential proteins that may interact with BCAR1. Among them, 68 genes were significantly positively correlated to BCAR1 expression, as verified by TCGA. Six hub genes were revealed by PPI String. High expression of POLR2A, MAPK3, MOV10, and XAB2 predicted poor prognosis in lung adenocarcinoma, as verified by the K‐M plotter database. POLR2A and MAPK3 are involved in both catalytic activity and transferase activity. POLR2A and BCAR1 were significantly increased in lung cancer tissues as compared with matched normal tissues. High expression of POLR2A was significantly positively correlated to BCAR1 overexpression and predicted poor prognosis in 54 lung cancer cases. POLR2A expression was significantly decreased following BCAR1‐KO in H1975 and H1299 cells.

**Conclusions:**

BCAR1 promotes proliferation and cell growth, probably via upregulation of POLR2A and subsequent enhancement of catalytic and transferase activities. However, additional robust studies are required to elucidate the mechanisms involved.

## Graphical abstract

In this work, we elaborated the enhancement effect of a novel carcinogenetic molecule, ie, p130cas (breast cancer antiestrogen resistance 1, BCAR1) on proliferation and cell growth of lung cancer cells,and to explore the possible networks of interacting proteins of BCAR1. We found BCAR1 can promote proliferation and cell growth, probably via upregulation of POLR2A and subsequent enhancement of catalytic activity and transferase activity.

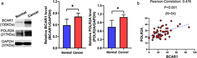

## Introduction

Breast cancer antiestrogen resistance protein 1 (BCAR1; Crk‐associated substrate, CAS; p130cas) has a modular structure. It includes an amino terminal domain, a substrate binding region, and a carboxy terminal domain. This is followed by a Src and phosphoinositide 3 kinase (PI3K) binding region, and is terminated by the C‐terminal homology domain (CCHD) of the CAS family.^1^, ^2^ Owing to this modular structure, the level of BCAR1 tyrosine phosphorylation largely depends on the binding capacity of its amino terminal domain. This allows BCAR1 to directly interact with the multiple protein motifs of various phosphatases and kinases, and to mediate Src through FAK bridge indirect association.

As an adaptor/scaffold protein, BCAR1 has been reported to be involved in the regulation of signal transduction, actin cytoskeleton remodeling, and the stability of cell structure, especially as a mechanically sensitive regulator of filamentous pseudopod stability.^2^, ^3^, ^4^, ^5^ Some oncogenes, such as ErbB2(erb‐b2 receptor tyrosine kinase 2), PTEN(phosphatase and tensin homolog), and PI3KCA(phosphatidylinositol‐4,5‐bisphosphate 3‐kinase catalytic subunit alpha) stimulate tumor progression via phosphorylation of BCAR1.^5^ BCAR1 has been shown to enhance tumor proliferation, invasion, and metastasis in several cancers. In prostate cancer, high expression of BCAR1 is closely related to early recurrence.^6^ In endometrial adenocarcinoma, BCAR1 is involved in both growth and metastasis.^7^ In oral squamous cell carcinoma, BCAR1 promotes bone invasion and metastasis by regulating epithelial‐mesenchymal transition (EMT) and cell proliferation.^8^ High expression of BCAR1 can alter breast epithelial cell morphology, which can lead to tumor formation as well as cell growth, invasion, and metastasis of breast cancer cells.^9^, ^10^

Our previous research demonstrated that high expression of BCAR1 in lung cancer promotes EMT, invasion, and metastasis of tumor cells, and it also predicts poorer prognosis in lung adenocarcinoma cases.^11^, ^12^, ^13^ Herein, we aimed to determine the role of BCAR1 in proliferation and cell growth in lung adenocarcinoma, on which few studies have previously focused. Furthermore, the networks of proteins that interact with BCAR1 to trigger the proliferation of lung cancer cells remain to be identified. Therefore, we designed this study to elucidate the abovementioned points.

## Methods

### Cell experiments

#### Cell culture

Human lung adenocarcinoma cell lines NCI‐H1975 (ATCC CRL‐5908) and NCI‐H1299 (ATCC CRL‐5803) and human embryonic kidney 293T cells (ATCC CRL‐3216) were cultured in RPMI‐1640 medium (Thermo‐Fisher,11875) supplemented with 10% fetal bovine serum (FBS; Gibco, 10099141) in a 5% CO_2_ incubator at 37°C.

#### CRISPR‐Cas9‐mediated knockout of BCAR1

**Figure 1:**
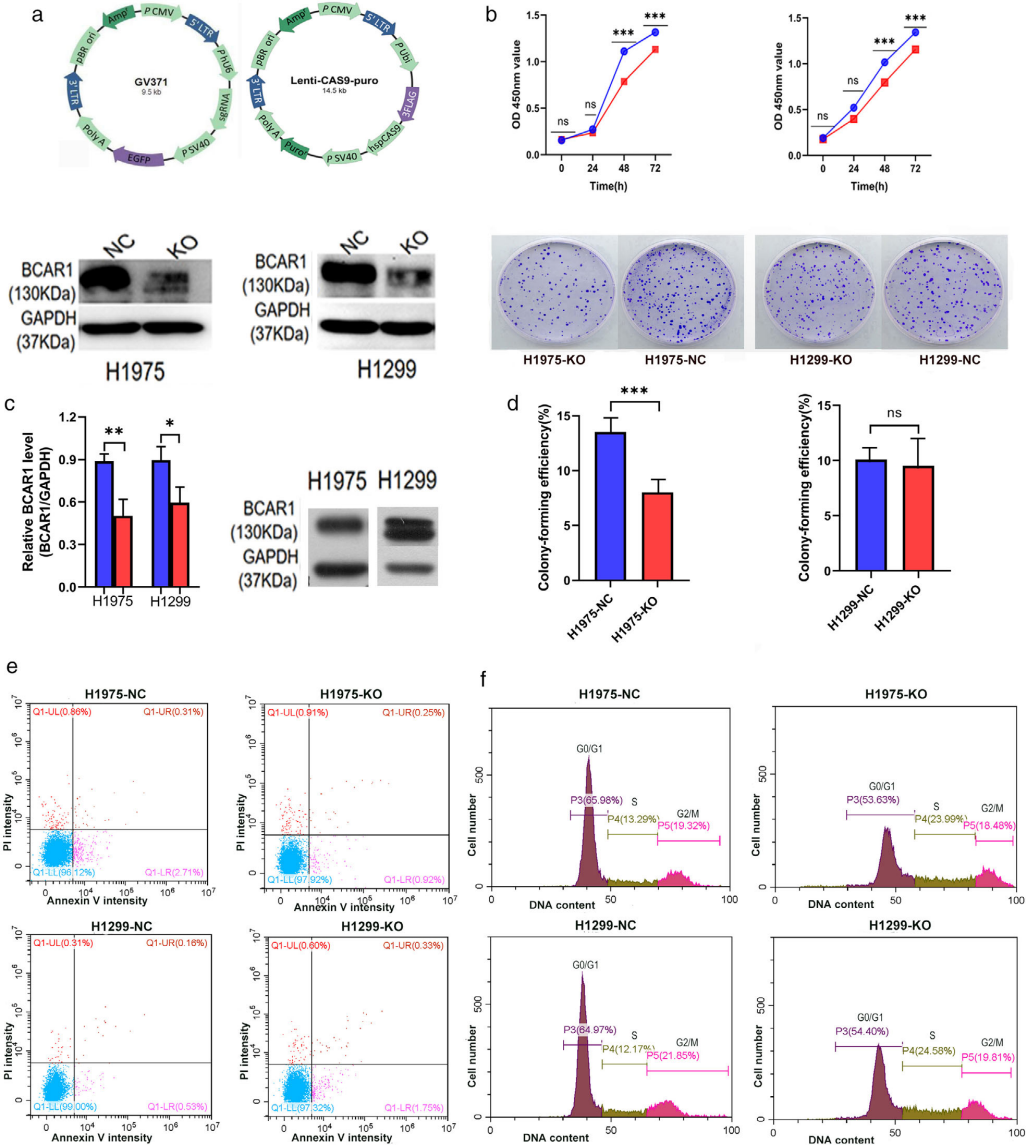
Cell proliferation, colony formation, apoptosis, and cell cycles of H1975 and H1299 cells after BCAR1 knockout. (**a**) CRISPR‐Cas9 system for BCAR1 knockout; (**b**) BCAR1 was found to be highly expressed in H1975 and H1299 cells, and BCAR1 was knocked out in either H1975 or H1299 cells (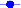) NC (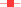) KO; (**c**) CCK‐8 assay showed proliferation of H1975 and H1299 cells was inhibited following BCAR1‐KO (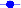) H1975NC (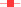) H1975KO (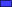) H1299NC (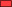) H1299KO; (**d**) Colony formation of H1975 cells was inhibited following BCAR1‐KO; however, there was no remarkable difference between BCAR‐KO and NC H1299 cells; (**e**) Flow cytometry indicated apoptosis did not differ following BCAR1‐KO; (**f)** Flow cytometry indicated cell cycles did not differ following BCAR1‐KO. (* *P* < 0.05; ** *P* < 0.01; and *** *P* < 0.001).

A stable knockout of BACR1 in H1975 and H1299 cells was achieved using a CRISPR‐Cas9 system. Briefly, we designed the interference target sg‐RNA according to the sequences of BCAR1 gene as follows: KO:CAGCAACGGGTCTCGGCCAT and NC:CGCTTCCGCGGCCCGTTCAA. The guide oligonucleotide was phosphorylated, annealed, and cloned into the BsmBI site of the GV371 vector (Jikai Gene Co., Ltd., Shanghai. P.R. China) as shown in Fig 1a. The constructed vector was then verified by sequencing. The transfer plasmid with the annealing guide oligonucleotide was transformed into *Escherichia coli* strain DH5α bacteria and the amplified plasmid was isolated from the bacteria using a plasmid isolation kit (MB001‐100420rnx, Gesesci Medical Technology Co., Ltd., Shanghai, P.R. China). Lentivirus was produced by transfecting 293T cells with transferable lenti‐CAS‐puro plasmid (Jikai Gene Co., Ltd., Shanghai, P.R. China), as shown in Fig 1a, and with packaging plasmids Helper 1.0 (Jikai Gene Co., Ltd., Shanghai, P.R. China) and Helper 2.0 (Jikai Gene Co., Ltd., Shanghai, P.R. China). The supernatant with virus was collected 48 and 72 hours after transfection and used to infect H1975 and H1299 cells, respectively. Then, 16 hours after infection, the medium containing lentivirus was replaced with fresh medium containing puromycin (2 μg/mL), and puromycin‐resistant cells were collected after seven days of selection. Finally, cell infection efficiency was confirmed by fluorescence microscopy and western blot. The detailed steps in a previously published protocol were followed.^14^

#### Western blot

BCAR1 and POLR2A were immunoblotted using anti‐BCAR1 (1:1000, CST, Inc., #13846S, USA) and anti‐POLR2A (1:500, ab817, abcam, Cambridge, MA, USA), respectively. An anti‐GAPDH (1:1000, CST, Inc., #2118, USA) antibody served as the control. A detailed experimental protocol is described in our previous study.^13^

#### Cell proliferation assay

Cell proliferation was examined using the Cell Counting Kit‐8 (Beyotime Biotechnology, C0037, USA) according to the manufacturer's protocols. Briefly, cells were seeded at a density of 5000 cells/well in 96‐well plates. CCK8 solution (10 μL) was added to each well 0, 24, 48 and 72 hours after seeding, and incubated for two hours. Finally, the absorbance was measured using a standard plate reader (Thermo Scientific Multiskan FC) at 450 nm.

#### Colony formation assay

In the logarithmic phase, the tumor cells were digested with 0.25% trypsin solution (Gibco, 25 200–056) to form a single cell suspension. Cells were then transferred to a 10 cm culture dish for cultivation at 1000 cells/well. The culture medium was changed according to the cell growth state. When cell colonies were clearly observed with the naked eye, the cells were no longer fed with medium. Cells were then fixed with 4% paraformaldehyde (Solarbio, P1110) and stained with 2.5% crystal violet (Solarbio, G1061‐10 ml) for 30 minutes.

#### Apoptosis and cell cycle assays

The Annexin V‐FITC/PI Apoptosis Detection kit (Beyotime Biotechnology, C1063) was used to detect apoptosis according to the manufacturer's instructions. Briefly, cells were digested with 0.25% trypsin without EDTM (Solarbio,T1350), centrifuged at 300 *g* for five minutes, and washed with PBS. Cells were then resuspended in 500 μL 1x annexin V binding buffer. Subsequently, 5 μL annexin V‐FITC and 5 μL PI were added to the buffer and incubated for 15 minutes in dark conditions at room temperature. Apoptosis was detected using flow cytometry (BD FACSCanto) within one hour.

The Cell Cycle Analysis Kit (Beyotime Biotechnology, C1052) was used to detect cell cycle progression according to the manufacturer's instructions. Briefly, cells were digested with 0.25% trypsin and centrifuged at 300 × *g* for five minutes. The cells were washed with ice‐cold PBS. Subsequently, 1 mL ice‐cold 70% alcohol was used to fix the cells at 4°C for 24 hours. The next day, cells were centrifuged at 300 × g for 5 minutes and then resuspended by adding 1 mL ice‐cold PBS and centrifuging again at 300 × g for 5 minutes. Finally, 0.5 ml proportioned dye solution (staining buffer, propidium iodide staining solution, RNase A) was added to each centrifuge tube. The cell cycle was detected by flow cytometry after 30 minutes in the dark.

#### Immunoprecipitation‐mass spectrometry (IP‐MS)

For BCAR1 overexpression construction and IP‐MS, 293T cells and GV141 vector^15^ (Jikai Gene Co., Ltd., Shanghai. P.R.China) were utilized. Primers for amplification of BCAR1 cDNA were designed and synthesized as follows: i. ACGGGCCCTCTAGACTCGAGCGCCACCATGCCTGCCAA.

GCCCTTCCTCTCTTC; and ii. AGTCACTTAAGCTTGGTACCGAGGCGGC.

TGCCAGCTGGCCTAGGAC. Subsequently, the nucleotides were inserted into GV141 (BCAR1‐OE) and verified by sequencing. Thereafter, GV141 (BCAR1‐OE) and negative control vector (empty GV141) were transfected into 293T cells using X‐tremeGENE HP DNA Transfection Reagent (Roche, 06366236001, Germany). Stable cells were selected at a sustained concentration of 200 μg/mL G418 (Solarbio,G8161) more than 24 hours after transfection.

The shotgun method was used for IP‐MS analysis. The main steps included protein extraction, filter aided proteome preparation digestion, mass spectrometry, and data processing. Briefly, 293T cells were used to extract proteins. SDT lysate (Beyotime Biotechnology,P0013) was added to the sample in a boiling water bath for 15 minutes and the sample was then centrifuged at 14000 × *g* for 15 minutes to obtain the supernatant. A BCA Protein Assay Kit (Beyotime Biotechnology, P0010S) was used for protein quantification. Subsequently, filter aided proteome preparation enzymatic hydrolysis was conducted using C18 Cartridge (Thermo Scientific Acclaim PepMap RSLC 50 μm × 15 cm, nanoviper, P/N164943) to desalt the peptide. Next, the peptide was lyophilized and 20 μL 0.1% formic acid solution (Thermo, A117) was added for reconstitution. When the sample was separated by chromatography, a Q Exactive mass spectrometer (Thermo Fisher Scientific) was used to perform mass spectrometry. The original data generated by Q Exactive was converted by Proteome Discoverer 2.2 (Thermo Fisher Scientific) software and submitted to MASCOT2.6 server (Matrix Science) for database searching. Then, the data formed on the MASCOT server were returned to the software via Proteome Discoverer 2.2. Ultimately, reliable results were obtained according to the standard of FDR <0.01. (FDR was calculated using the Benjamini‐Hochberg method).

### Bioinformatic analysis

The protein atlas database (https://www.proteinatlas.org/) was used to evaluate the expression of BCAR1 across a spectrum of cell lines, including lung adenocarcinoma cells.

Enrichment analysis of molecular functions were performed by Gene Ontology (GO), powered by PANTHER (http://geneontology.org/).

The search conditions and steps in The Cancer Genome Atlas (TCGA; https://www.cancer.gov/)^16^ database were as follows: (i) Cancer type: LUAD; (iia) Search dataset: RNASeq; (iib) Stage I; (iii) Attribute gene: BCAR1; (iv) Target dataset: LUAD RNASeq; and (v) Statistical method: Pearson correlation analysis. The genes with expression that was significantly positively correlated to BCAR1 expression were yielded by setting FDR ≤10–6 as the end point.

The online PPI STRING software (version 11.0, https://string-db.org/cgi/input.pl) was used to analyze potential networks of BCAR1 as per the published protocols.^17^

The predictive power of gene expression in the prognosis of lung adenocarcinoma was evaluated by the K‐M plotter database (https://kmplot.com/analysis/).^18^ To exclude confounders of survival, cohorts were limited to early stage cases.

### Validation by clinical specimen

#### Patients

The study protocol was reviewed and approved by the Research Ethics Board in Daping Hospital (Chongqing City, P.R. China) (TMMU‐DPH), and informed consent was written and obtained from all patients. In the study performed from November 2015 to April 2019, there were a total of 54 patients with early stage lung adenocarcinoma. Pulmonary neoplasm was diagnosed radiographically and confirmed by pathology. None of the patients had received treatment prior to enrollment in the study. The demographic and clinicopathological characteristics of patients are shown in Table 1.

**Table 1:** Clinical and demographical characteristics of 54 lung adenocarcinoma cases in early stage

Variables	Number of cases
Gender (male: female)	24:30
Age (year)	60.4 ± 9.1
Tumor size (cm)	2.3 ± 0.7
Stage (1a:1b)	46:8
Survival time (days)	582.5 ± 385.9

Postoperative follow‐up was completed for 54 cases by telephone or letter interview.

#### Tissue samples

We obtained tissue proteins by homogenizing cancer tissues and paired adjacent normal tissues of five lung adenocarcinoma patients. Western blots detected expression of BCAR1 and POLR2A.

#### Construction of tissue microarray (TMA)

Tissue microarray fabrication methods were performed as previously described^3^ .

#### Immunohistochemistry (IHC) experiment

TMA was used for further IHC experiments. The sections were incubated with serum blocking solution and primary antibodies, including p130cas antibody (CST, #13846S, 1:100), POLR2A antibody (Abcam, ab817, 1:100), and corresponding secondary antibodies SignalStain Boost IHC Detection Reagent (HRP, Rabbit, CST, #8114) and Goat Anti‐Mouse IgG H&L (Alexa Fluor 488, Abcam, ab150113). Next, diaminobenzidine solution was used as a chromogen and slides were counterstained in a hematoxylin solution. The average optical density of IHC protein detection was calculated using Image J software (1.52a version, Wayne Rasband, National Institutes of Heath, USA).

#### CO‐IP assays

Cell protein was extracted and BCAR1 antibody was coated on agarose beads to immunoprecipitate the BCAR1 protein. The immunoprecipitate was separated by denaturation and the BCAR1 (P130, Santa Cruz, SC‐20029,1:500) and POLR2A proteins (Abcam, ab817, 1: 500) were analyzed by western blot.

### Statistical analysis

The comparison of measured data was performed using student's *t*‐test or paired‐samples *t*‐test for statistical analysis. Pearson correlation was used for continuous variables. The prognostic factors were examined by univariate and multivariate analysis using the Cox proportional hazard model. K‐M plotter was carried out for prognostic survival analysis. All of these calculations were performed using IBM SPSS Statistics 26.0 software (Chicago SPSS, USA). *P* < 0.05 (two‐sided) was considered statistically significant.

## Results

### Proliferation, colony formation, apoptosis, and cell cycles of H1975 and H1299 cells after BCAR1‐KO

#### BCAR1 expression across cell lines

As shown in Fig S1, BCAR1 was highly expressed in a variety of cell lines. These cells lines included endothelial cells from veins and skin, breast cancer cells, colon and liver cancer cells, and lung cancer cells. Herein, we chose to study preclinical and clinical implications of BCAR1 in lung adenocarcinoma.

#### Stable knockout of BCAR1 in both H1975 and H1299 cells

BCAR1 was found to be highly expressed in both H1975 and H1299 cells (Fig 1b). Therefore, we chose to perform experiments in vitro using these cells. As shown in Fig 1b, BCAR1 expression in the KO group was significantly lower than that of the negative control (NC) group in H1975 and H1299 cells, suggesting that BCAR1 knockout was successful (0.50 ± 0.11 vs. 0.89 ± 0.05, *P* < 0.01 and 0.60 ± 0.11 vs. 0.90 ± 0.10, *P* < 0.05).

#### BCAR1 knockout inhibits proliferation of H1975 and H1299 cells

Following BCAR1‐KO, proliferation of H1975 and H1299 cells was significantly inhibited after 48 hours and 72 hours compared to NC, (Fig 1c; H1975 at 48 hours: 0.787 ± 0.032 vs. 1.110 ± 0.103, *P* < 0.001; H1975 at 72 hours: 1.132 ± 0.152 vs. 1.317 ± 0.176, *P* < 0.001; H1299 at 48 hours: 0.797 ± 0.145 vs. 1.015 ± 0.173, *P* < 0.001; H1299 at 72 hours: 1.157 ± 0.019 vs. 1.345 ± 0.076, *P* < 0.001).

#### BCAR1‐KO inhibits colony formation of H1975 cells

As shown in Fig 1d, colony‐formation efficiency of H1975 cells was significantly decreased following BCAR1‐KO, compared to NC (8.03% ± 0.51% vs.13.53% ± 0.55%, *P* < 0.001). However, there was no significant difference between BCAR‐KO and NC in H1299 cells (9.53% ± 1.77% vs. 10.1% ± 0.53%, *P* = 0.623).

#### BCAR1‐KO did not change apoptosis or cell cycle progression

As shown in Fig 1e, there was no difference in apoptosis of H1975 and H1299 cells following BCAR1‐KO. Similarly, as shown in Fig 1f, there was no difference in the cell cycle of H1975 and H1299 cells following BCAR1‐KO.

### IP‐MS and bioinformatic analysis predict potential interactions with BCAR1

#### Potential proteins may interact with BCAR1

**Figure 2:**
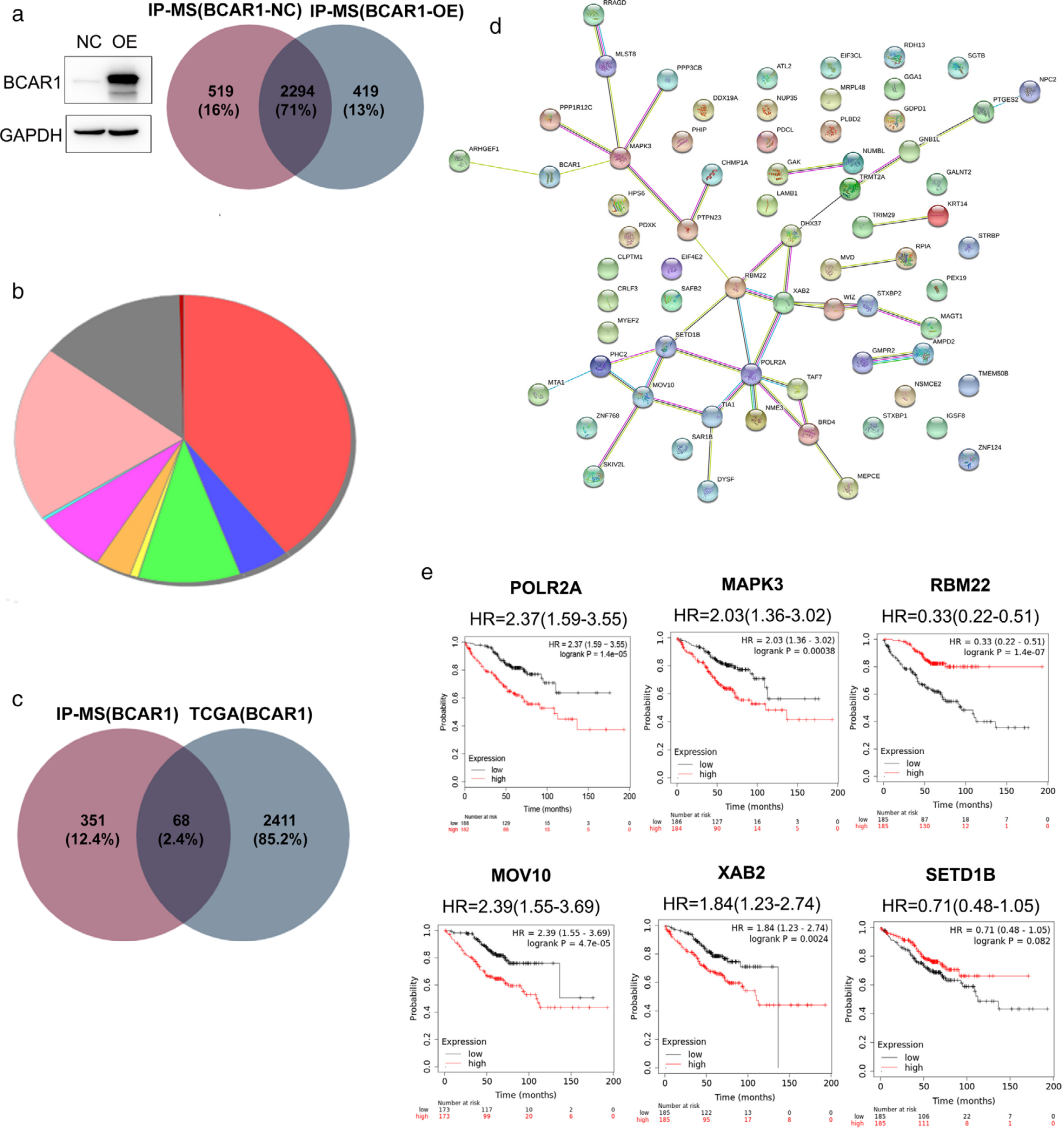
Bioinformatics analysis. (**a**) BCAR1 was overexpressed in 293T cells and IP‐MS demonstrated 419 potential proteins that may interact with BCAR1; (**b**) Enrichment analysis of molecular functions of 419 potential proteins (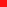) catalytic activity(GO:0003824) (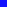) catalytic activity, acting on RNA(GO: 0140098) (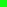) kinase activity(GO:0016301) (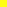) molecular transducer activity(GO:0060089) (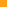) nucleotidyltransferase activity(GO:0016779) (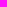) protein serine/threonine kinase activity(GO:0004674) (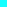) signaling receptor activity(GO:0038023) (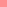) transferase activity(GO:0016740) (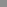) transferase activity, transferring phosphorus‐containing groups(GO:0016772) (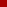) transmembrane signaling receptor activity(GO:0004888); (**c**) 68 genes were significantly positively correlated to BCAR1 expression and verified by TCGA; (**d**) Potential networks of interacting proteins of BCAR1 revealed by PPI String software; (**e**) Prognostic power of six hub genes in lung adenocarcinoma evaluated by K‐M plotter database.

As shown in Fig 2a, Western blot assays demonstrated that overexpression of BCAR1 in 293T cells was successfully achieved (0.1989 ± 0.07185 vs.1.385 ± 0.03043, *P* < 0.001). By IP‐MS assay, 2713 proteins were pulled down by overexpressed BCAR1 that was labeled by FLAG (BCAR1‐OE). Among them, 2294 proteins were also pulled down by FLAG (BCAR1‐NC) as shown in Fig 2a . Ultimately, 419 proteins were identified that may interact with BCAR1 (Fig 2a and Table S1).

#### Catalytic activity and transferase activity of BCAR1 interaction partners

As shown in Fig 2b, enrichment analysis of 419 genes showed that catalytic activity and transferase activity were the top two molecular functions, performed by 101 and 50 genes, respectively. There were 50 genes involved in both functions, including (RNA polymerase II subunit A) POLR2A and (mitogen‐activated protein kinase 3) MAPK3 (Table S2).

#### TCGA verification of positive correlation to BCAR1 expression

TCGA demonstrated that 2479 genes were significantly positively correlated to BCAR1 expression. Among them, there was 68 genes which were overlapped with the abovementioned 419 genes that were verified by IP‐MS (Fig 2c).

#### PPI String to reveal hub genes

Upon analyzing the abovementioned 68 genes via PPI String (Fig 2d), we obtained six genes which had at least four links. These genes included POLR2A (7 links), MAPK3 (5 links), RNA‐Binding Motif Protein 22(RBM22; 5 links), SET Domain Containing 1B (SETD1B; 4 links), Mov10 RISC Complex RNA Helicase(MOV10; 4 links), and XPA binding protein 2 (XAB2; 4 links).

#### High expression of POLR2A, MAPK3, MOV10 and XAB2 predicted poor prognosis in lung adenocarcinoma

As shown in Fig 2e, the K‐M plotter database indicated that high expression of POLR2A, MAPK3, MOV10, and XAB2 predicted poor prognosis in lung adenocarcinoma. However, RBM22 and SETD1B showed the opposite trend. Intriguingly, POLR2A and MAPK3 were included in 50 genes which were involved in both catalytic activity and transferase activity (Table S2), demonstrating critical molecular functions. Since POLR2A was linked to more genes than the other three hub genes, suggesting more possibility of involvement in a signaling cascade, we focused on POLR2A for further verification.

### POLR2A correlation with BCAR1 and survival prediction

#### POLR2A was significantly positively correlated to BCAR1 overexpression and predicted poor lung cancer prognosis

**Figure 3:**
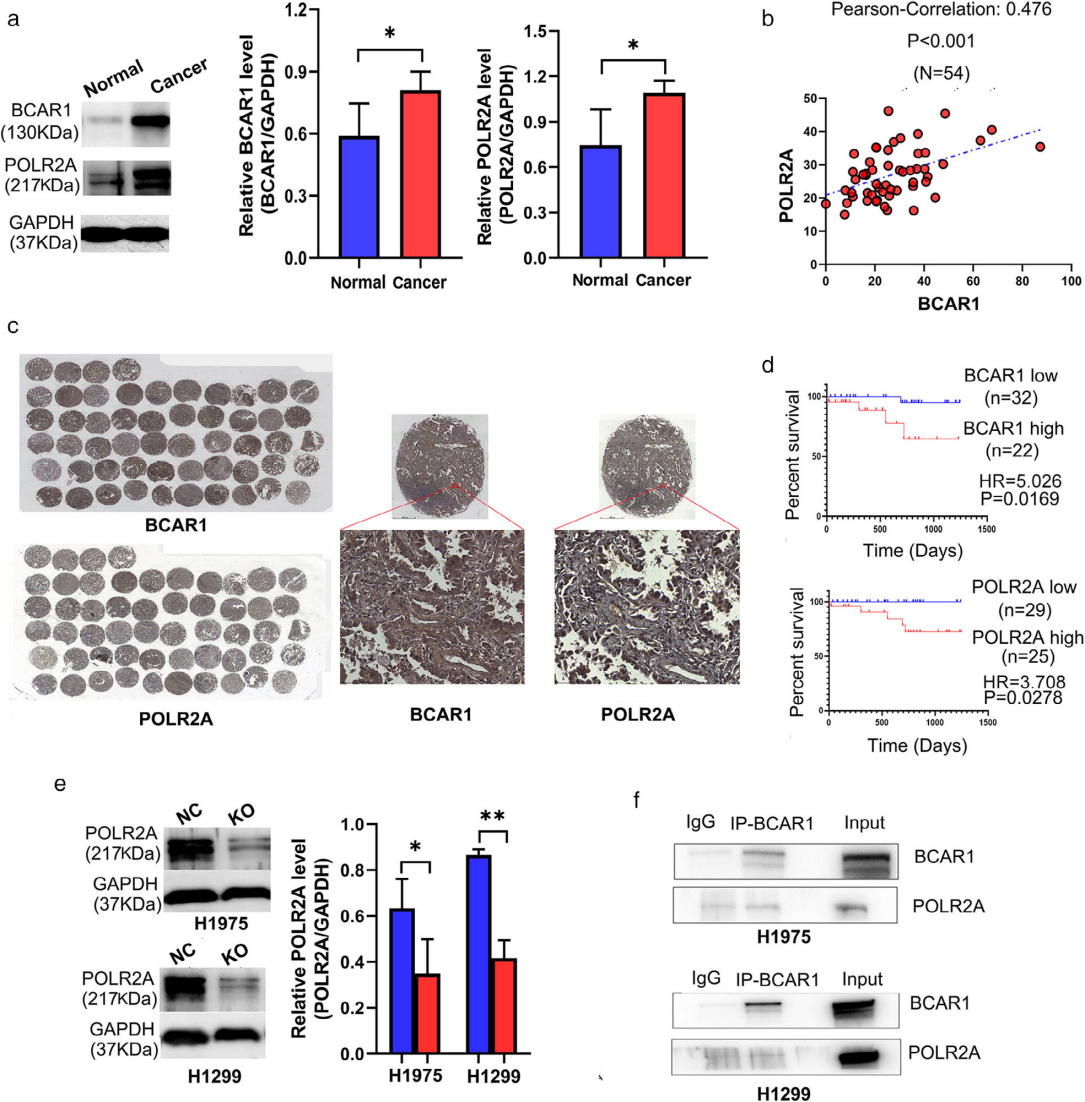
The relationship between BCAR1 and POLR2A as well as their prognostic significance in lung adenocarcinoma. (**a**) Western blot revealed that POLR2A and BCAR1 expression was significantly increased in lung cancer tissues compared to adjacent normal tissues; (**b**) IHC‐stained TMA showed that BCAR1 is expressed in nucleus, cytoplasm, and both; however, POLR2A is highly expressed in nucleus; (**c**) POLR2A expression was significantly positively correlated to BCAR1 expression in lung cancer tissues; (**d**) High expression of either BCAR1 or POLR2A predicted poor prognosis of 54 lung cancer cases in early stage; (**e**) Western blot showed POLR2A expression was significantly decreased following BCAR1‐KO (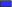) NC (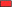) KO; (**f**) Co‐IP assay revealed BCAR1 cannot pull down POLR2A in H1975 and H1299 cells (* *P* < 0.05; ** *P* < 0.01; and *** *P* < 0.001).

As shown in Fig 3a, POLR2A and BCAR1 were significantly increased in lung adenocarcinoma tissues compared to adjacent normal tissues (1.090 ± 0.082 vs. 0.746 ± 0.236, *P* < 0.05 and 0.810 ± 0.090 vs. 0.589 ± 0.156, *P* < 0.05, respectively).

IHC‐stained TMA are shown in Fig 3b and demonstrate that BCAR1 was expressed in the nucleus, in the cytoplasm, or in both locations. However, POLR2A was highly expressed in nucleus. As shown in Fig 3c, POLR2A expression was significantly positively correlated to BCAR1 expression (R = 0.476, *P* < 0.001). Neither BCAR1 nor POLR2A expression was correlated with tumor size (data not shown). High expression of either BCAR1 or POLR2A predicted poor prognosis in 54 lung cancer cases in early stage (Fig 3d and Table 2).

**Table 2:** High BCAR1 or POLR2A expression predicted poor prognosis

Risk factor	*P*‐value	HR	95% CI lower/upper
Tumor size	0.242	2.817	0.498 15.940
BCAR1	0.001	5.026	1.948 12.963
POLR2A	0.015	3.708	1.290 10.655

CI, confidence interval; HR, Hazard ratio.

#### Decreased POLR2A in H1975 and H1299 cells following BCAR1‐KO

As shown in Fig 3e, western blot analysis demonstrated that POLR2A was significantly decreased in BCAR1‐KO compared to NC cells (H1975: 0.416 ± 0.043 vs. 0.633 ± 0.127, P < 0.05 and H1299: 0.416 ± 0.079 vs. 0.865 ± 0.024, *P* < 0.01). This suggests that BCAR1 regulates POLR2A in H1975 and H1299 cells, despite the negative results of the CO‐IP (Fig 3f).

## Discussion

Studies have confirmed that BCAR1 is involved in carcinogenetic processes such as cell survival, growth, invasion, and migration. Moreover, BCAR1 has been considered as a new potential molecular marker for lung cancer.^19^ High expression of BCAR1 predicts poor prognosis in lung cancer patients^11^, ^12^, ^20^ and is associated with lymph node metastasis, distant organ metastasis,^4^, ^20^ and chemotherapy resistance in lung cancer.^4^ Furthermore, BCAR1 is found to be highly expressed in lung cancer cell lines.^11^ Cell growth arrest, inhibition of cell migration, and EMT can occur following BCAR1‐KO in lung cancer cells.^13^, ^20^

However, to our knowledge, no study has previously investigated the role of BCAR1 in lung cancer cell proliferation. Moreover, the proteins that interact with BCAR1 remain unclear. In this study, we first established BCAR1‐KO cells in H1975 and H1299 cell lines using CRISPR‐CAS9. After knockout of BCAR1, H1975 and H1299 cell proliferation was remarkably inhibited, and H1975 cell colony formation was also significantly decreased. There was no significant difference in colony formation in H1299 cells, probably due to lower efficacy of BCAR1 knockout in these cells. BCAR1‐KO had no effect on cell apoptosis, in accordance with our previous study.^11^ Intriguingly, BCAR1‐KO in lung adenocarcinoma cell lines had no impact on the cell cycle. This was in discordance with a previously published study, which showed that the FAK/p130cas/Rac signaling pathway promoted cell cycle progression in mouse embryo fibroblasts.^21^ We hypothesize that BCAR1 may play different biological roles in various cell types.

IP‐MS combined with TCGA analysis was used to screen the potential interacting proteins of BCAR1. Subsequently, we employed the online String software to predict potential BCAR1 signaling pathways. Fortunately, we obtained four hub genes (MAPK3, MOV10, XAB2, and POLR2A), which potentially interact with BCAR1 and play carcinogenetic roles in lung cancer.

MAPK3 is a kinase that has various biological functions, including promoting cell growth, cell proliferation, and cell cycle progression in a variety of cancers.^22^, ^23^, ^24^, ^25^ MOV10 is regarded as an important regulator of the nervous system, which promotes angiogenesis of glioma cells by regulating cell activity, migration, and tube formation.^26^ Abnormal expression of MOV10 is related to radiotherapy resistance and cell proliferation in breast cancer.^27^ XAB2 is a protein with multiple cell functions, which are mainly integrated into various processes such as DNA splicing, DNA damage repair, DNA transcription, and mRNA output.^28^ XAB2 can inhibit the activity of TP53 in lung cancer cell lines, which indirectly promotes the occurrence of cancer.^29^ Considering POLR2A had the most links among the four hub genes in the PPI string, we focused on POLR2A for further verification and validation.

**Figure 4:**
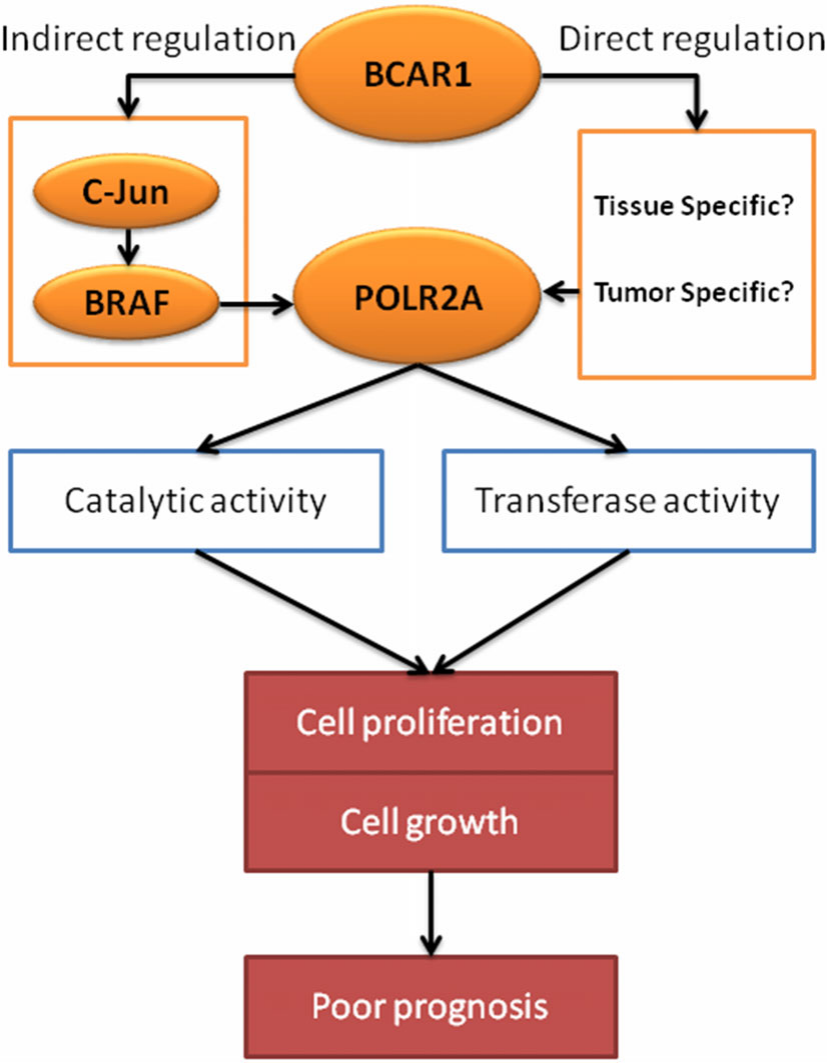
Speculation of carcinogenetic roles of BCAR1 via upregulation of POLR2A.

POLR2A is the largest subunit encoding RNA polymerase II and is combined with other polymerase subunits to form the DNA binding domain of polymerase. The POLR2A gene contains a carboxy‐terminal domain composed of heptad repeats, including serine and threonine residues. The main biological function of POLR2A is to guide the synthesis of mRNA.^30^ Moreover, there has been an increasing number of studies on the effects of POLR2A on tumors. High expression of POLR2A predicts poor prognosis in breast cancer patients, and selective targeting of POLR2A can inhibit cell growth.^31^, ^32^ Downregulation of POLR2A expression in colorectal cancer inhibited the proliferation and survival of tumor cells.^33^, ^34^ Therefore, POLR2A is promisingly expected to become a new target for colorectal cancer or breast cancer treatment. The SNP (rs2071504 C) and SNP (c.2292C > T, rs2228130) in POLR2A was associated with poor survival outcomes in lung cancer,^35^, ^36^ suggesting the potential carcinogenic role of POLR2A in lung cancer. In this study, POLR2A was found to be involved in the enhancement of catalytic activity and transferase activity, which has been shown to promote cell proliferation and cell growth.^37^, ^38^, ^39^ Herein, we did not find a direct interaction between BCAR1 and POLR2A in H1975 and H1299 cells. POLR2A was pulled down by BCAR1 in 293T cells, probably due to tissue‐specific or tumor‐specific interactions between these two proteins. However, the BCAR1/c‐JUN cascade has been shown to upregulate the expression of BRAF,^40^ which is believed to be an upstream regulator of POLR2A.^41^ Based on these findings, we speculate that BCAR1 can, at the very least, indirectly upregulate POLR2A expression, which has critical biological functions leading to cell proliferation, cell growth, and subsequent poor prognosis in lung cancer cases (Fig 4).

The current study is not without limitations. The underlying mechanism of how BCAR1 and POLR2A are connected remains unclear. Moreover, synergism analysis is needed to demonstrate the role of interaction between BCAR1 and its partners with respect to proliferation and cell growth in lung cancer. Future robust studies are required to resolve the interesting abovementioned points.

## Disclosure

The authors declare that there is no conflict of interest.

## Supporting information


**Figure S1** BCAR1 expression across cell lines

**Table S1** Supplementary Tables
