# Supplementary figures and images for: BCAR1 promotes proliferation and cell growth in lung adenocarcinoma via upregulation of POLR2A
Source: Thorac Cancer. 2020 Oct 1;11(11):3326–36. doi: 10.1111/1759-7714.13676 (PMC7606008; doi:10.1111/1759-7714.13676)

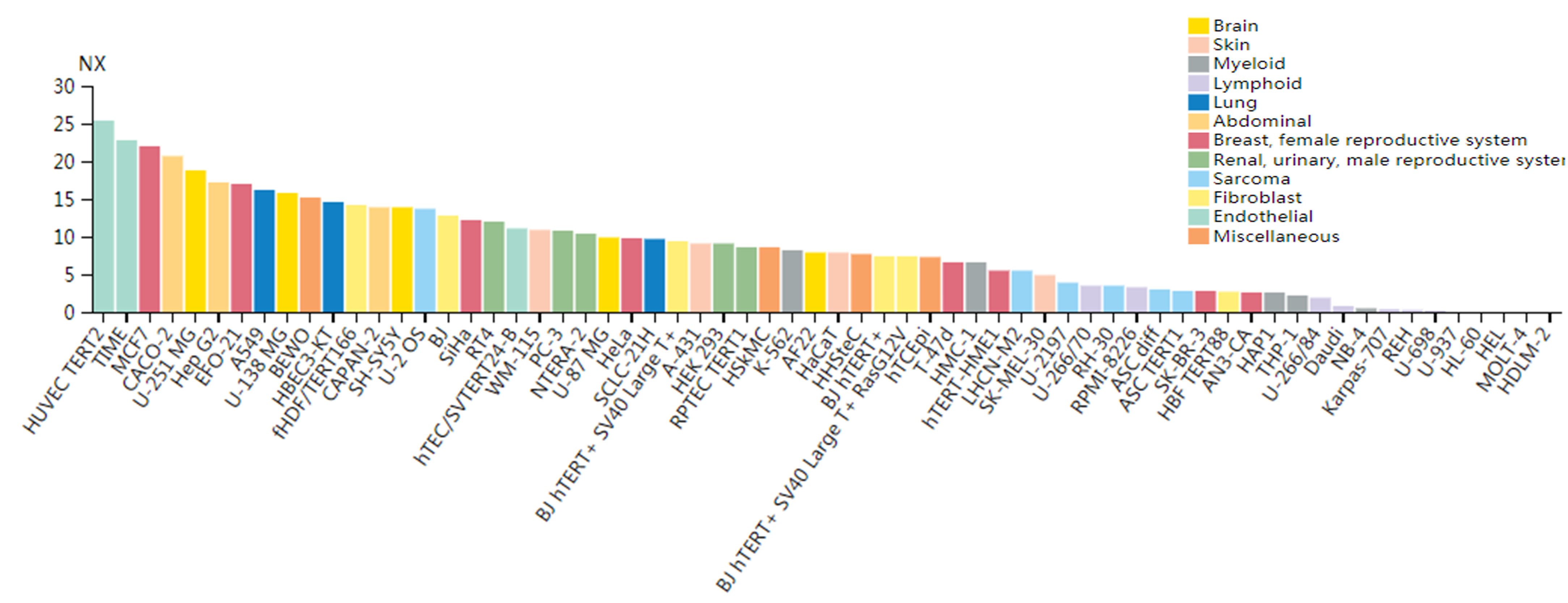

Supplement: Supplementary file 1 — Figure S1 BCAR1 expression across cell lines [file TCA-11-3326-s001.tif]
